# Role of Gender on the Outcomes of ST-Elevation Myocardial Infarction Patients Following Primary Coronary Angioplasty

**DOI:** 10.7759/cureus.17892

**Published:** 2021-09-11

**Authors:** Aigin Heydari, Aryan Zahergivar, Peyman Izadpanah, Gilberto Aquino, Jeremy R Burt

**Affiliations:** 1 Cardiology, Shiraz University of Medical Sciences, Shiraz, IRN; 2 Radiology, Medical University of South Carolina, Charleston, USA; 3 Cardiothoracic Imaging, Medical University of South Carolina, Charleston, USA

**Keywords:** stemi, outcome, gender, primary coronary angioplasty, st-elevation myocardial infarction

## Abstract

Background

There are considerable differences in the prevalence of coronary artery disease (CAD) and its cardiovascular risk factors between men and women. Due to the significance of gender as a factor that potentially affects cardiovascular disorders and patient outcomes, the present study aimed to assess the baseline characteristics and outcomes of CAD patients in terms of gender distribution.

Methods

All consecutive patients diagnosed with ST-elevation myocardial infarction (MI) who had undergone primary percutaneous coronary intervention (PCI) in the previous two years in a comprehensive cardiology center were included. Data were retrospectively collected from the hospital record files. Color Doppler echocardiography, valvular involvement, and the type of coronary vessel involvement were also evaluated.

Results

In total, 557 consecutive patients (437 men and 120 women) were included with a mean age of 59.37 ± 26.23 years and 64.07 ± 11.60 years for men and women, respectively (p = 0.004). The prevalence of mitral regurgitation (MR) and tricuspid regurgitation (TR) was significantly higher among women than men.

Conclusion

Female patients who suffered from CAD and underwent PCI were older than men. Also, ischemic mitral regurgitation (MR) and tricuspid regurgitation (TR) were more prevalent among women, while smoking was more prevalent among men.

## Introduction

ST-segment elevation myocardial infarction (STEMI) is considered the main cause of morbidity for decades worldwide [1]. Of note, ischemic heart disease has become the first cause of death and disability-adjusted life years (DALYs) during recent years in Iran [2]. Therefore, ongoing research and trials are being conducted to determine the associated risk factors for further risk management. Modifying lifestyle patterns would facilitate the path to improved cardiovascular risk profiles as well as avoiding revascularization [3].

Myocardial revascularization is the restoration of blood flow in stenosed or occluded coronary arteries through invasive strategies such as coronary artery bypass grafting (CABG) and percutaneous coronary intervention (PCI) [4]. Both procedures can effectively restore blood flow in the native coronary arteries, leading to the revitalization of ischemic cardiac tissues. In this context, the preferred strategy of reperfusion within 1.5-2 hours from the first medical contact is PCI (door-to-balloon time = 90 minutes) [4,5]. Although the PCI strategy has been highly successful with minimal post-procedural complications even in high-risk groups [4,6], many other factors affect the overall outcome.

In this regard, ongoing surveys are produced to determine the relevant factors and provide plenary guidelines based on individuals’ characteristics. In this context, gender has shown a prominent role in the ultimate outcomes of STEMI patients who undergo PCI. The results vary in different parts of the world [7-9]. Herein, we investigate the risk and importance of assessing the outcomes of PCI including post-interventional ejection fraction (EF) and valvular disease for primary treatment of STEMI based on gender as a risk factor for patients in our region.

## Materials and methods

In this retrospective survey, we studied 557 consecutive patients who had undergone primary PCI following the diagnosis of STEMI in a cardiology center affiliated to Shiraz University of Medical Sciences (SUMS), Shiraz, Iran, from January 2017 to February 2018 (Al-Zahra Heart Hospital). The mentioned hospital is the main center of PCI in Shiraz. Among a total of 1328 total cases of PCI, patients with a history of previous myocardial infarction (MI), previous PCI, or incomplete records were excluded, and finally, 557 patients met all the inclusion criteria. We curated the following demographic data from the patients’ hospital records: smoking history, length of hospital stay, in-hospital mortality, color Doppler echocardiography results which was obtained 48 hours after the PCI, including the valvular involvement and ejection fraction (EF), and type and number of coronary vessel involvement. The study was approved by the Ethics Committee of SUMS (Ethic code no. IR.SUMS.MED.REC.1398.069). Informed consent was waived by the ethics committee.

Statistical analysis

Descriptive analysis was used to describe the data, including mean ± standard deviation (SD) for quantitative variables and frequency (percentage) for categorical variables. The correlation between quantitative variables was assessed using Pearson's or Spearman's correlation test. To determine the gender difference in the study variables, a multivariable logistic regression model was employed. The Chi-squared test, t-test, and Mann-Whitney test were used to compare variables. For these analyses, we used IBM SPSS Statistics for Windows version 23.0 (IBM Corp., Armonk, USA). P-values below 0.05 were considered to be statistically significant.

## Results

**Figure 1:**
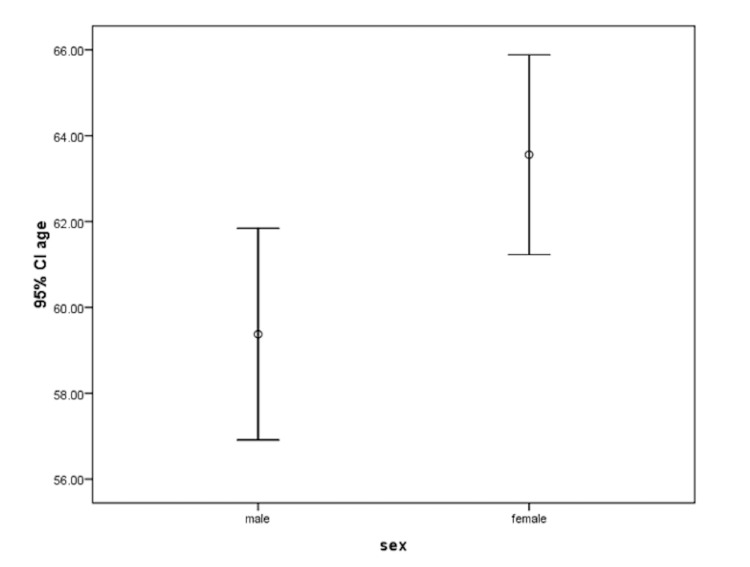
Mean age in men and women

In total, 557 consecutive patients (437 [78.4%] men and 120 [21.6%] women) were included in this study (Table 1). The mean age of men and women was 59.3 ± 26.2 and 64.0 ± 11.6 years, respectively (p = 0.004) (Figure 1). In total, 277 (63.4%) men and 28 (23.3%) women were smokers (p < 0.001).

**Table 1:** Baseline characteristics of the study participants MR = mitral regurgitation; TR = tricuspid regurgitation; AI = aortic insufficiency; MS = mitral stenosis; LAD = left anterior descending coronary artery; RCA = right coronary artery; LCX = left circumflex coronary artery; LM = left main coronary artery; LVEF: left ventricular ejection fraction

Item	Men (N= 437)	Women (N=120)	P-value
Mean age, years	59.37 ± 26.23	64.07 ± 11.60	0.004
Current Cigarette smoking, %	277 (63.4%)	28 (23.3%)	< 0.001
Mean LVEF, %	41.36 ± 9.49%	40.61 ± 10.29	0.449
MR	238 (54.5%)	78 (65.0%)	0.039
TR	114 (26.1%)	43 (35.8%)	0.036
AI	15 (3.4%)	7 (5.8%)	0.286
MS	3 (0.7%)	0 (0.0%)	N/A
LAD	707 (92.4%)	110 (91.7%)	0.776
RCA	299 (68.4%)	88 (73.3%)	0.301
LCX	289 (66.1%)	75 (62.5%)	0.459
LM	6 (1.4%)	3 (2.5%)	0.110
One-vessel	89 (20.4%)	26 (21.7%)	N/A
Two-vessel	140 (32.0%)	33 (27.5%)	N/A
Three-vessel	208 (47.6%)	61 (50.8%)	N/A

**Figure 2:**
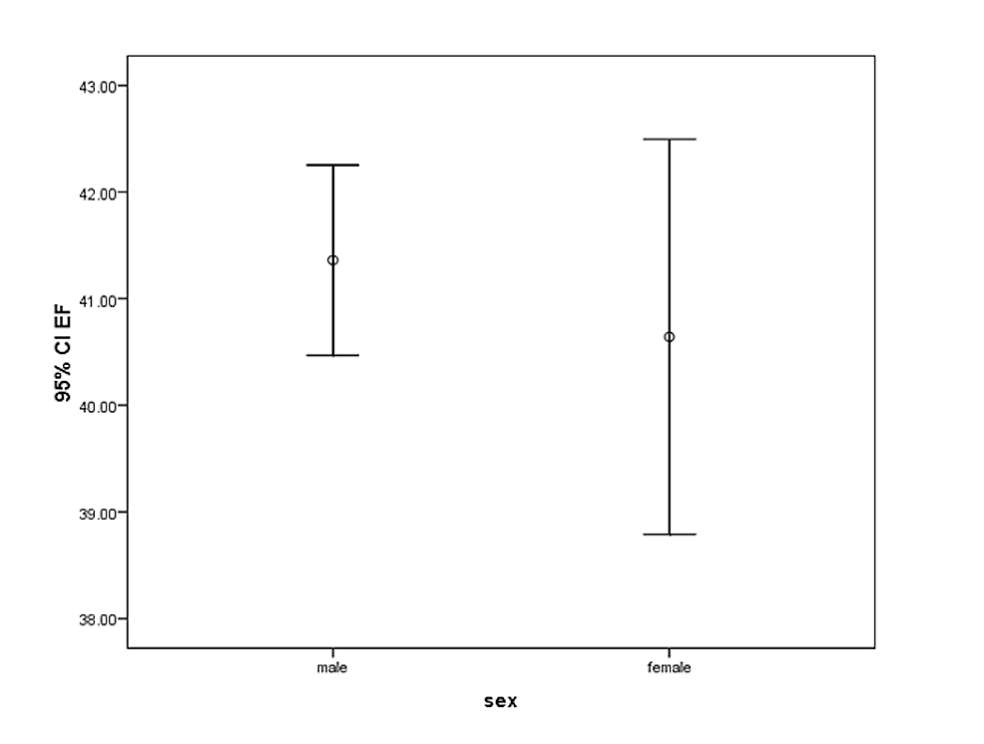
Mean LVEF in men and women LVEF: left ventricular ejection fraction

**Figure 3:**
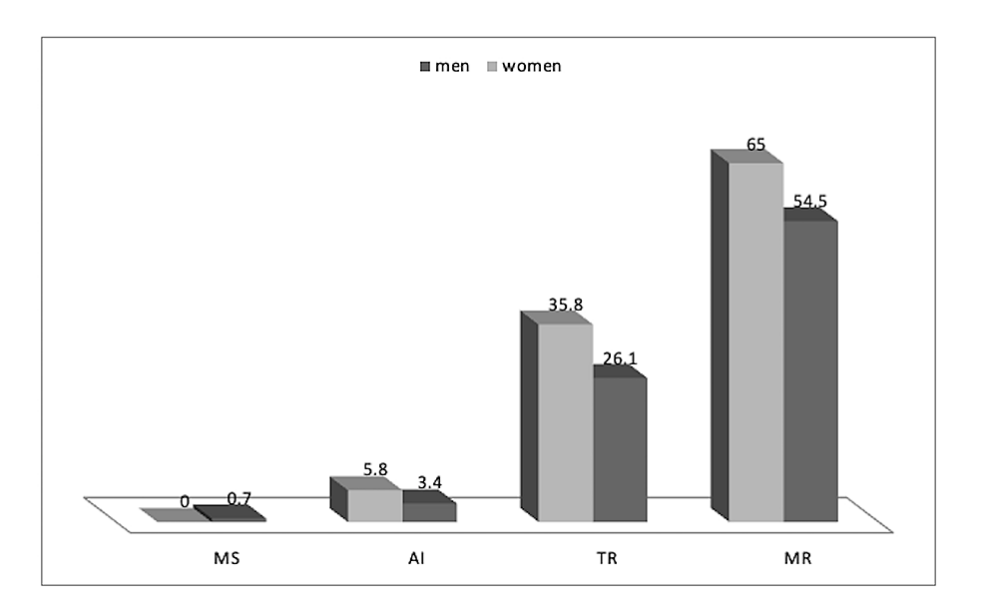
Valvular involvement in men and women MS: mitral stenosis; AI: aortic insufficiency; TR: tricuspid regurgitation; MR: mitral regurgitation

The mean left ventricular ejection fraction (LVEF) which was measured in the post-PCI transthoracic echocardiography (TTE) in men and women was 41.36 ± 9.49 and 40.61 ± 10.29 percent, respectively (p = 0.449) (Figure 2). Concerning valvular defects, the prevalence of ischemic mitral regurgitation (MR) was 238 (54.5%) and 78 (65.0%) amongst men and women, respectively (p = 0.039). In the same order, the prevalence of ischemic tricuspid regurgitation (TR) was 114 (26.1%) and 43 (35.8%) (p = 0.036), ischemic aortic regurgitation (AR) was 15 (3.4%) and 7 (5.8%) (p = 0.286), and mitral stenosis (MS) was 3 (0.7%) and 0 (0.0%), respectively. A significant difference was found between men and women in terms of the prevalence of ischemic MR and TR (Figure 3). Of note, smoking was more prevalent among men.

**Figure 4:**
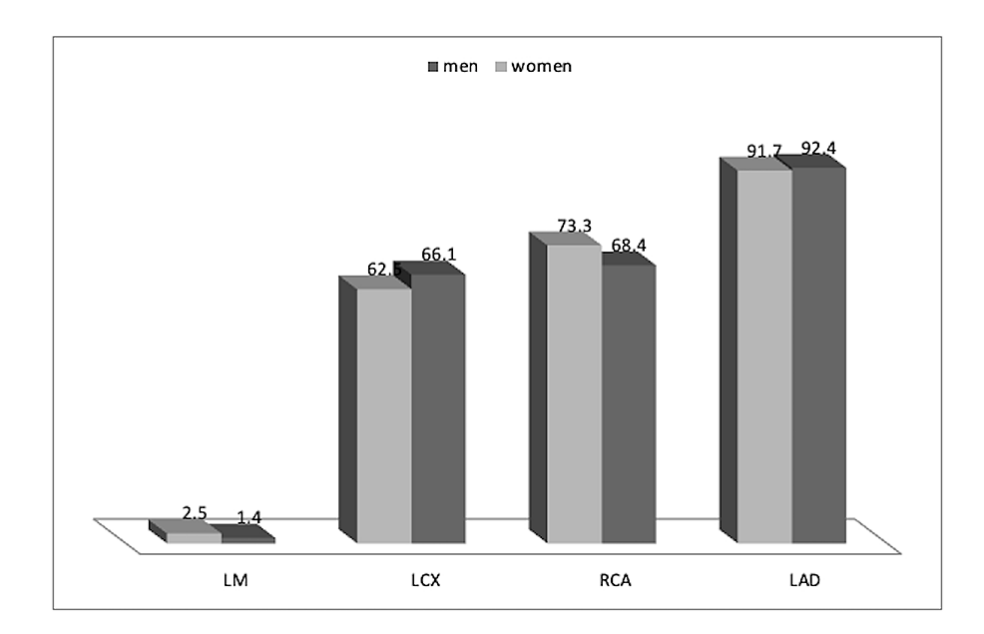
Type of coronary vessel involvement in men and women

Concerning the coronary vessels involved in men and women, the left anterior descending (LAD) artery was involved in 404 (92.4%) and 110 (91.7%) cases (p = 0.776), the right coronary artery (RCA) in 299 (68.4%) and 88 (73.3%) (p = 0.301), the left circumflex (LCX) in 289 (66.1%) and 75 (62.5%) (p = 0.459), and the left main (LM) in six (1.4%) and three (2.5%) (p = 0.110), respectively. Hence, no significant differences between men and women were seen (Figure 4). In terms of the number of vessels involved in men and women, one-vessel involvement was detected in 89 (20.4%) and 26 (21.7%), two-vessel involvement in 140 (32.0%) and 33 (27.5%), and three-vessel involvement in 208 (47.6%) and 61 (50.8%), respectively, with no significant difference between men and women (p = 0.636).

Procedure-related death occurred in 14 (3.2%) men and 6 (5.0%) women, where no significant difference was detected (p = 0.349) (Table 2). The need for CABG was reported in 52 (11.9%) men and 14 (11.7%) women, showing similarity between the two genders (p = 0.938). The CABG procedure in this population was finally done within 30 days of their angiography results. The average hospital stay in men and women was 4.37 ± 2.21 and 4.38 ± 1.85 days, respectively (p = 0.882).

**Table 2:** PCI-related outcomes in the male and female participants of this study PCI: percutaneous coronary intervention; CABG: coronary artery bypass graft

Item	Men (N=)	Women (N=)	P-value
Death	14 (3.2%)	6 (5.0%)	0.349
Need for CABG	52 (11.9%)	14 (11.7%)	0.938
Length of hospital stay (“in days”)	4.37 ± 2.21	4.38 ± 1.85	0.882

Gender was not a significant determinant for PCI-related death when adjusted for baseline variables (OR = 0.552, p = 0.705) (Table 3). Also, in a multivariable linear regression model, gender was not a predictor of the length of hospital stay for PCI (beta = -0.812, p = 0.417) (Table 4).

**Table 3:** Multivariate logistic regression model in the assessment of the effect of gender on PCI-related death Hosmer-Lemeshow: Chi-square = 8.432, p = 0.392 EF: ejection fraction; MR: mitral regurgitation; TR: tricuspid regurgitation

Variable	P-value	OR	95.0% CI for OR	
			Lower	Upper
Sex	0.552	0.705	0.223	2.231
Age	0.164	0.992	0.980	1.003
Cigarette	0.833	1.120	0.390	3.215
EF	0.001	1.136	1.083	1.192
MR	0.979	1.015	0.336	3.064
TR	0.821	0.880	0.290	2.672
Vessels	0.428	0.759	0.383	1.503
Constant	0.806	1.534		

**Table 4:** Multivariate logistic regression model in assessment of gender effect on PCI-related death R square = 0.045 EF: ejection fraction; MR: mitral regurgitation; TR: tricuspid regurgitation

Model		Unstandardized Coefficients		Standardized Coefficients	t	P value
		B	Std. Error	Beta		
1	(Constant)	4.824	0.676		7.133	0.000
	sex	-0.188	0.231	-0.036	-0.812	0.417
	age	0.002	0.004	0.028	0.658	0.511
	cigarette	0.307	0.191	0.072	1.607	0.109
	EF	-0.026	0.009	-0.116	-2.707	0.007
	MR	-0.109	0.203	-0.025	-0.536	0.592
	TR	-0.229	0.223	-0.048	-1.029	0.304
	VESSELS	0.346	0.115	0.127	3.022	0.003

## Discussion

Due to considerable differences in the overall prevalence of CAD and related cardiovascular risk factors between men and women as well as the significant effect of gender on the likelihood of cardiovascular disorders, the present study aimed to assess baseline characteristics and outcomes of patients undergoing PCI for primary treatment of STEMI using a sample of Iranian men and women. As the main findings, we found notable differences in age, current smoking rate, and prevalence of MR and TR across the two genders. However, there was no difference in the state of left ventricular function (assessed by LVEF) and in the number of coronary arteries involved between men and women. Regarding outcomes, gender did not affect the CAD-related mortality rate, need for repeated revascularization, or hospital stay. Baseline cardiovascular status and post-PCI complications were not affected by gender. The results were also confirmed by adjusting baseline characteristics. It seems that the rate of CAD complications after revascularization depends on sex, genetic, racial, and environmental factors. While some authors demonstrated similar findings to our survey, others expressed contradictory results. In general, female sex has been linked to a poorer prognosis following coronary revascularization, with a higher risk of death and MI in women undergoing PCI. This has been attributed to older age, higher prevalence of comorbidities, and stronger coronary artery disease (CAD) risk profile [10-12]. Although women less than 50 years of age are at lower risk for developing CAD, they may be at higher risk for adverse events once diagnosed, thereby representing a subgroup of patients at increased risk for adverse cardiovascular events [13].

It seems that the discrepancy between the two genders in the outcomes of revascularization should be adjusted for baseline variables - especially age. As indicated in our survey, the similarity between men and women remained even after adjustment for the age factor. However, some previous reports are inconsistent with our findings. In a study by Argulian et al. in 2006 [14], women were more likely to be older, with a greater prevalence of hypertension and diabetes mellitus compared with men. After adjusting for baseline characteristics and coronary artery size, the incidence of coronary vascular injury complications was higher in women than in men, particularly among the young. No significant gender differences were present in the combined endpoint of death, myocardial infarction, and emergency CABG surgery, which is completely similar to our observation. In a study by Epps et al. in 2016 [15], although procedural success rates were similar by gender, the cumulative rate of major adverse cardiovascular events was higher in young women, driven largely by higher rates of repeat revascularizations, which is contrary to our findings. In another study by Guo et al. in 2018 [16], the in-hospital mortality in male patients was significantly lower than those of females. The major adverse cardiovascular events (MACE) decreased significantly in male subjects after initial PCI compared with females. In another study by Heer et al. in 2017 [17], there were no sex-related differences in in-hospital mortality among patients undergoing PCI, but access-related complications were twice as high in women, irrespective of the indication. In a study by Cenko et al., the female sex was associated with post-procedural Thrombolysis in Myocardial Infarction (TIMI) flow grade 0 to 2 and higher mortality [18]. In a study by Josiah et al. in 2018 [19], there was no significant gender difference in the number of vessels attempted, the mean number of lesions treated, or the mean number of stents used. On multivariate analysis, the female sex was not a predictor of death, and there was no significant gender difference in the overall incidence of unadjusted 1-year MACE. In another study by Gudnadottir et al. in 2017 [20], all in-hospital complications following PCI were more frequent among women. There was no gender difference in adjusted 30-day mortality after PCI or CABG. Finally, Worrall-Carter et al. in 2017 [21] showed that compared to men, women were older at admission, less likely to be diagnosed with STEMI, and less likely to smoke, but no gender difference was observed for severe co-morbidities or the use of coronary angiography.

Well explained in the literature, there is a wide spectrum of findings between men and women in terms of age on admission, CAD severity before PCI, initial left ventricular function, and early outcomes after PCI. These variations might be due to differences in the type of study planning, study power, racial and genetic characteristics of study populations, and the time of following up.

Our study had some remarkable strengths, particularly a great number of subjects. We faced some limitations as well; the study was retrospective so we had no hand in determining the cases or interacting with the procedures. The detailed demographics of the patients (e.g. BMI, previous history of hypertension and hyperlipidemia, pack-year index, etc) were not available for the whole population, so the study was limited to the factors applicable to all the patients.

## Conclusions

According to our study, women suffering from STEMI and undergoing primary treatment with PCI are older than men. Also, ischemic MR and TR are more prevalent in women as compared to men. Regarding the outcomes of PCI, there are no differences in procedural death, left ventricular systolic function, need for CABG, and length of hospital stay between the two genders. A multi-center prospective study is warranted to validate our results.
